# The effect of oral contraceptives on orthodontic tooth movement in rat

**DOI:** 10.4317/medoral.18048

**Published:** 2012-12-10

**Authors:** Pooya Olyaee, Behnam Mirzakouchaki, Kavoos Ghajar, Seyyed A. Seyyedi, Majid Shalchi, Alireza Garjani, Esmaeil Dadgar

**Affiliations:** 1 DDS, MSc, Assistant Professor, Department of orthodontics, Faculty of dentistry, Urmia University of Medical Sciences, Urmia, Iran; 2 DDS, MSc, Associate Professor, Department of orthodontics, Faculty of dentistry, Tabriz University of Medical Sciences, Tabriz, Iran; 3 DDS, Resident of Endodontics, HamedanUniversity of medical sciences, Hamedan, Iran; 4 DDS, MSc, Assistant Professor, Department of oral medicine, Faculty of dentistry, Urmia University of Medical Sciences, Urmia, Iran; 5 DDS, MSc, Assistant Professor,Guilan University of medical sciences, Rasht, Iran; 6 PhD, Professor, Faculty of Pharmacy, TabrizUniversity of Medical Sciences; 7 DDS, Private practice, Tabriz, Iran

## Abstract

Introduction: The purpose of this study was to investigate the effect of ethinyl estradiol/norgestrel � used in some oral contraceptives- on orthodontic tooth movement in Wistar rats. Material and Methods: Forty eight female three-month old Wistar rats with an average weight of 250�25gr were divided into two experimental and control groups. One week prior to appliance insertion and during the appliance therapy period, 100 mcg/kg/day of ethinyl estradiol and 1mg/kg/days of norgestrel were administered to the experimental group by gavage; meanwhile the control group received an equivalent volume of Sodium Chloride 0.9 % (Saline). Maxillary central incisors were tipped distally by insertion of springs exerting 30g force. Two, seven and fourteen days after spring insertion animals were sacrificed. The mesioincisal distance between maxillary incisors were measured. Subsequently, histological sections were prepared for histomorphometric studies. Results: 14 days after force application the orthodontic tooth movement was significantly lower in the experimental group (p<0.05). The number of osteoclasts were significantly lower in the experimental group 2, 7 and 14 days after spring insertion (p<0.05). Conclusion: Ethinyl estradiol/norgestrel (oral contraceptives) can significantly decrease the amount of tooth movement in the linear phase.

** Key words:**Oral contraceptives, orthodontic tooth movement, ethinyl estradiol, norgestrel.

## Introduction

Orthodontically induced tooth movement is the normal result of applying a mechanical force to a tooth. In the general view of orthodontic tooth movement, bone formation is associated with the tension side and resorption with the compression side. Three fundamental biologic responses are attributed to orthodontic loads: bone formation, bone resorption, and iatrogenic external root resorption (1).

Remodeling consists of the removal, by osteoclasts, of a quantumof bone followed by the formation by osteoblasts, resulting in tooth movement. Many systemic hormones influence bone modeling and remodeling. In addition to the sex steroids, these include parathyroidhormone (PTH), thyroid hormones, growth hormone, glucocorticoids,and 1,25(OH)2D. Many of these act via the production of locallyproduced factors and may also interact with mechanical stimulito affect bone modeling andremodeling (2).

Oral contraceptive pills (OCP) apply an association of synthetic estrogen (usually ethinyl estradiol) and a progestagen (3). This is the most frequently used method for contraception in adolescents (4). Studies carried out on the effect of oral contraceptives, with different progestagen components, on bone turnover, have shown that contraceptives reduce bone turnover. In the most recent study, Vescovi et al. (5) demonstrated that 2 weeks of low-dose oral contraceptive therapy rapidly reduced markers of bone resorption and formation.

Regarding the composition of oral contraceptives, it seems possible that OCP consumption affects tooth movement via changes in bone turnover. The purpose of the present study is to investigate the effect of an oral contraceptive including ethinyl estradiol/norgestrel on orthodontic tooth movement in a rat model.

## Material and Methods

A total of forty eight 3-month-old female Wistar rats, with an average weight of 250�25gr, were used in this study. Animals were examined in two groups randomly; experimental (n=24) and control (n=24).Each group was divided into three subgroups of 8 each.

Animals were acclimatized for 7 days in plastic cages (two per cage) with a standard 12-hour light/dark cycle. The temperature was maintained at 25� C and humidity at 50%. Animals were fed with a diet of finely ground laboratory food ad libitum, and had access to drinking water. The rats were anesthetized with an intraperitonal injection of ketamine (50 mg/kg) before spring insertion.

One week prior to appliance insertion, and during the appliance therapy period the medication was administered to the experimental group 5 days a week (except Thursday, and Friday) by gavage. The daily doses included 100 mcg/kg ethinylestradiol (Estinyl�, Aboureihan, Tehran, Iran) plus 1 mg/kg norgestrel (Ovrette�, Aboureihan, Tehran, Iran), in a 0.5% carbocxy methyl cellulose suspension.The control group was administered an equivalent volume of saline (Sodium Chloride 0.9%, Aboureihan, Tehran, Iran).

Orthodontic force was administered to all groups to distalize maxillary central incisors. Springs were bent from 0.35-mm stainless steel wire (Dentaurum, Germany) with a length of 8 millimeters, modified in the light of the previous studies by Mirzakouchaki and Firoozi (6).The springs were placed on a grid and activated on a single arm with pliers. The force was measured with a gauge (ETM, America). Springs were attached by means of the bands (Dentaurum, Germany), and a 30 g reciprocal force was applied to the teeth. The springs were not reactivated during the experiment.

The distance between the mesial corners of the maxillary incisors was measured by each author before appliance insertion with a digital caliper with accuracy of 0.01 mm. The appliance was placed on maxillary central incisors using an orthodontic band seater, and glass ionomer cement (Bandtite, America). The bands had a 3 mm distance with the incisal edge of the teeth, incisocervically. A thin spacer (0.05 mm) was placed between the central incisors before cement setting to prevent adhesion of the centrals to each other.

Two, seven and fourteen days after appliance insertion, 8 rats from each group (experimental and control) were randomly sacrificed by overdose of anesthetic drug. The distance between the mesial corners of maxillary central incisors was measured.

After sacrificing the rats, their premaxillae were dissected and placed in 10% formalin for four days. After fixation, the springs were removed, and the premaxillae were decalcified with 5% nitric acid for two days. The decalcified premaxillae were fixed again in the same manner for another three days. The sample was then dehydrated in a graded series of ethanol and embedded in paraffin (passage process).Then processes of clearing and impregnation were carried out. The paraffin blocks were sectioned serially using a microtome (Leitz, Germany), at 5 micrometer intervals in the frontal plane.

Five serial sections, each 5 �m thick were obtained 400 �m away from the alveolar crest. The sections, including maxillary central incisors (left and right), their alveolar bone and nasal septum were obtained perpendicular to the roots of the teeth. The sections were mounted on glass microscope slides and stained with hematoxylin and eosin. The osteoclasts were counted in a 1mm2 surface under a light microscope. Osteoclasts were identified as cells situated at the bone surface and containing more than three nuclei, a large amount of cytoplasm, and location in a resorption cavity. The Institutional Ethics Committee of Tabriz Medical University granted ethical clearance and the study followed the Helsinki guidelines prescribed for animal experimentation.

The data were analyzed by SPSS 14 software (SPSS for Windows, SPSS, Chicago, USA).Descriptive statistics are given as means�SEM. Smirnov-Kolmogrov test was employed to investigate normal distribution. Independent sample t-test was used to compare the amount of tooth movement, and osteoclast number between experimental and control group (p<0.05 is considered significant). One- way ANOVA, and Tukey test for post hoc was used to compare the amount of tooth movement and osteoclast number between 2nd, 7th and 14th day after appliance therapy in each group.

## Results

After employing Kolmogrov-Smirnov test the data showed normal distribution.

1- The distance between mesial corners of maxillary central incisors (amount of tooth movement) after two days of appliance therapy was 0.77�0.04 in the control group and 0.78�0.13 in the experimental group. Independent sample t-test showed that there was no significant difference in the amount of tooth movement between control and experimental group after two days.

2- The amount of tooth movement after seven days of appliance therapy was 1.00�0.17 in the control group and 0.74�0.04 in the experimental group. There was no significant difference in the amount of tooth movement between control and experimental group after seven days.

3- The amount of tooth movement after fourteen days of appliance therapy was 1.64�0.17 in the control group and 1.00�0.09 in the experimental group. There was a significant difference in the amount of tooth movement between control and experimental group after fourteen days (p=0.007).

4- The amount of tooth movement after two, seven and fourteen days of appliance therapy was 0.77�0.04, and 1.00�0.17 and 1.64�0.17 respectively in the control group ( Table 1). ANOVA test revealed that there was a significant difference in the mean amount of tooth movement in the control group(F(2,21)=9.53, p=0.001).Tukey (post hoc) test showed that there is a significant difference in the amount of tooth movement between days two and fourteen (p=0.001), and days seven and fourteen (p=0.15).

**Table 1:**
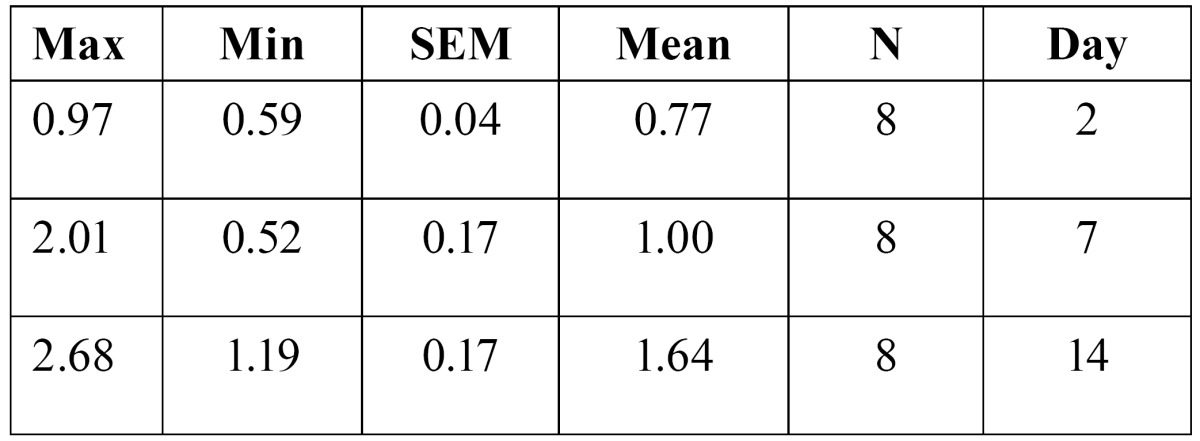
The amount of tooth movement in control group.

5- The amount of tooth movement after two, seven and fourteen days of appliance therapy was 0.78�0.13, and 0.74�0.04 and 1.00�0.09 respectively in the experimental group ( Table 2). ANOVA test revealed that there was no significant difference in the mean amount of tooth movement in the experimental group.

**Table 2:**
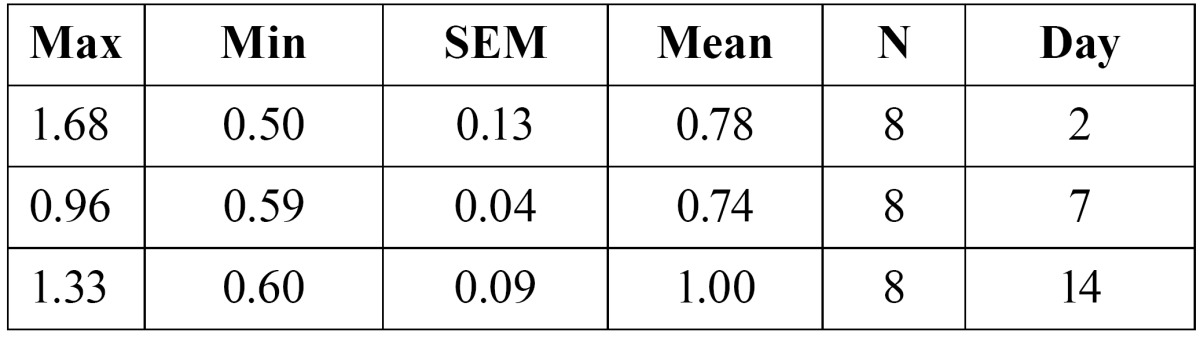
The amount of tooth movement in experimental group.

6- The mean number of osteoclasts per square millimeter of microscopic field on the movement side (osteoclast number) two days after appliance therapy was 1.37�0.15 in the control group and 0.86�0.09 in the experimental group. Independent sample t-test revealed that there was a significant difference between control and experimental group (p=0.018).

7- Osteoclast number seven days after appliance therapy was 2.69 �0.41 in the control group and 1.51 �0.19 in the experimental group. There was a significant difference between control and experimental group (p=0.029).

8- Osteoclast number fourteen days after appliance therapy was 3.15 �0.15 in the control group and 1.70 �0.14 in the experimen-tal group. There was a significant difference between control and experimental group (p<0.0005).

9- Osteoclast number after two, seven and fourteen days of appliance therapy were 1.37�0.15, and 2.69�0.41 and 3.15�0.15 respectively in the control group ( Table 3). ANOVA test revealed that there was a significant difference in the mean amount of tooth movement in the control group(F(2,21)=11.43, p<0.0005). Tukey (post hoc) test showed that there is a significant difference in the amount of tooth movement between days two and seven(p=0.007), and days two and fourteen(p<0.0005).

**Table 3:**
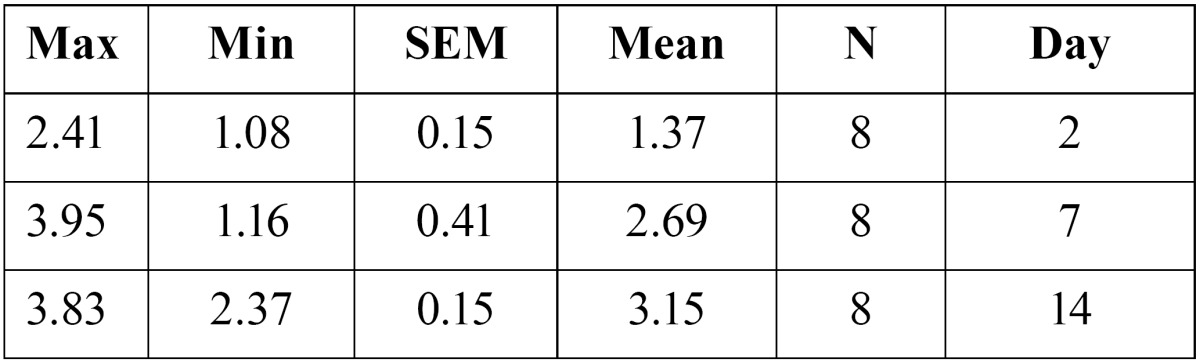
Osteoclast number in the control group.

10- Osteoclast number after two, seven and fourteen days of appliance therapy was 0.86�0.09, and 1.51�0.19 and 1.70�0.14 respectively in the experimental group ( Table 4). ANOVA test revealed that there was a significant difference in the mean amount of tooth movement in the control group(F(2,21)=8.18, p=0.002).Tukey (post hoc) test showed that there is a significant difference in the amount of tooth movement between days two and seven(p=0.019), and days two and fourteen(p=0.002).

**Table 4:**
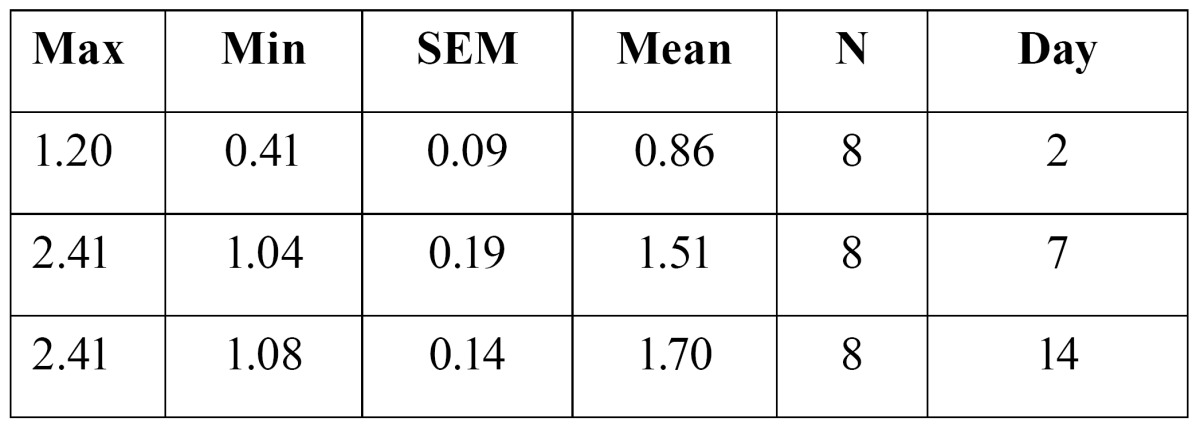
Osteoclast number in the experimental group.

## Discussion

In the present study administration of ethinyl estradiol/norgestrel (oral contraceptive) caused decreased tooth movement, and especially a significant decrease in the rate of tooth movement was observed fourteen days after force application (21 days after drug administration). Ethinyl estradiol/norgestrel also resulted in a significantly decreased number of osteoclasts at the movement side two, seven, and fourteen days after force application.

The time-course of tooth movement is known to have the following three characteristic phases in rats: instantaneous tooth movement as the first phase, delayed tooth movements the second one, and a linear increment of tooth movement as the third one (7).

In the present study, instantaneous tooth movement phase was observed during the first two days and could be due to viscoelastic properties of the periodontal tissues in the first stage (8). No significant difference was noticed in the amount of tooth movement two days after force application between control and experimental groups; this may be because the amount of tooth movement during the instantaneous phase is due to viscoelastic properties of the periodontal ligament. The delayed tooth movement phase was observed between the third and seventh days after force application. There was also no significant difference between the amounts of tooth movement seven days after force application. Thereafter, a linear increment of tooth movement was observed in our study, and the alveolar bone remodeled with a balanced sequence of bone formation and resorption in this phase (9). There was no significant effect of ethinyl estradiol/norgestrel on the rate of tooth movement during the instantaneous and delayed tooth movement phases; however, it was of interest that ethinyl estradiol/norgestrel significantly decelerated tooth movement during the linear phase. This finding suggests that tooth movement in each phase was regulated differently, and the specific effect of ethinyl estradiol/norgestrel on orthodontic tooth movement could be associated with oral contraceptive-induced bone metabolic changes. This process seems to be time dependent. Future studies are needed to investigate how long does it take for contraceptives to reduce alveolar bone turnover, and consequently tooth movement in human beings.

The present appliance was designed to produce a continuous horizontal force, and caused a tipping movement of the teeth. Once the appliance had been adjusted to produce 30 gram of force before installation, no activation was necessary during experimental tooth movement, and no deformity of the appliance was noted in any of the experimental rats at its removal. Tipping movement of the teeth results in pressure and tension at the periodontal ligament. It has been confirmed that the periodontal ligament is stretched and compressed at tension and pressure sites, respectively, 24 hours after force application. However, when the tooth movement shows a linear increment the width of the PDL is almost constant. Moreover, similar activation of both bone formation, and resorption at pressure sites is observed and confirmed by histomorphometry. It has been suggested that an increased rate of experimental tooth movement could activate bone remodeling in a coupled sequence of formation, and resorption (7).

Oral contraceptives have been shown to reduce bone turnover, but to our knowledge, no previous studies have been done on the effect of oral contraceptives on alveolar bone turnover. Farish et al. reported a marked reduction in bone turnover and decrease in bone resorption within the first month of oral contraceptive administration (10). Milner et al. (11), showed that alkaline phosphates- a marker of bone turnover- was significantly reduced in cases taking ethinyl estradiol/norgestrel. Sultana et al. (12) also showed that oral contraceptive users had lower level of mean serum alkaline phosphatase and higher mean bone mineral density. In the latest study by Vescovi et al. (5), it has been demonstrated that two weeks of oral contraceptive therapy rapidly reduces markers of bone resorption and formation.

In the present study osteoclast number at the movement side was selected as a quantitative index of alveolar bone remodeling. Histomorphometric analysis showed a significantly decreased number of osteoclasts at the movement side, two, seven, and fourteen days after force application. This finding is in agreement with the previously mentioned studies which show reduction in bone remodeling after oral contraceptive consumption. Osteoclasts are primarily observed two days after force application. We noticed a significantly lower number of osteoclasts two days after appliance insertion in the experimental group. This decrease in osteoclast number may be due to one week administration of oral contraceptive prior to appliance insertion. It has been suggested that maximum osteoclast recruitment happens five to seven days after force application (13). In the present study osteoclast recruitment was significantly lower seven days after force application and also during the linear phase after fourteen days. These findings show a reduced rate of bone remodeling at the movement side, after administration of ethinyl estradiol/norgestrel.

Estrogen inhibits bone remodeling by concurrently suppressing osteoblastogenesis and osteoclastogenesis from marrow precursors. Estrogen inhibits bone resorption via effects on the RANKL/RANK/osteoprotegerin system, as well as by reducing the pro-duction of a number of pro-resorptive cytokines (such as IL-1, IL-6), along with direct effects on osteoclast activity and lifespan (14). Relatively little is known about the effects of progestins on bone metabolism. In the ovariectomised rat model,progesterone was reported to have similar effects to estrogenin one study (15), but antagonistic actions in another (2). In a review by Thijssen, it has been concluded that there are no indications that the various progestins, used in clinical practice, have either a bone-protective or an estrogen antagonistic activity (16). Progestins do not add or subtract much of the protective action of estrogens on the bones (16). During experimental tooth movement in the present study, ethinyl estradiol/norgestrel resulted in a significant decrease in the number of osteoclasts. In our opinion this effect is mostly because of the estrogenic component; ethinyl estradiol.

## Conclusion

In the present study it was observed that administration of ethynil estradiol/norgestrel in Wistar rats significantly reduced orthodontic stooth movement, 14 days after appliance insertion, and osteoclasts at the movement side. Therefore, female patients should be informed that oral contraceptives may reduce their orthodontic tooth movement and subsequently increase treatment period.
